# Exosome release and neuropathology induced by α-synuclein: new insights into protective mechanisms of Drp1 inhibition

**DOI:** 10.1186/s40478-019-0821-4

**Published:** 2019-11-19

**Authors:** Rebecca Z. Fan, Min Guo, Shouqing Luo, Mei Cui, Kim Tieu

**Affiliations:** 1 Department of Environmental Health Sciences, Florida International University, Miami, USA; 2 Peninsula Schools of Medicine and Dentistry, Plymouth University, Plymouth, UK; 3 Department of Neurology, Huashan hospital, Fudan University, Shanghai, China

**Keywords:** Parkinson’s disease, Mitochondrial dynamics, Neurodegeneration, Alpha-synuclein, Autophagy, Protein aggregation

## Abstract

Targeting alpha-synuclein (α-syn) as a therapeutic strategy for Parkinson’s disease (PD) has been intensively pursued largely due to its well-recognized pathogenic role. Since its discovery as the first familial link to PD over two decades ago, this protein has been associated with multiple neurotoxic mechanisms, such as mitochondrial dysfunction and impaired autophagic flux. We report here that blocking dynamin-related protein 1 (Drp1) improved both mitochondrial function and autophagic flux in experimental models of α-syn. Using rat dopaminergic neuronal cells with inducible wild-type human α-syn, we observed excessive mitochondrial fragmentation and increased Drp1 levels 48 h after gene induction. Functionally, these cells exhibited lower mitochondrial membrane potential, reduced ATP production rate and mitochondrial spare respiratory capacity, as well as increased levels of mitochondrial reactive oxygen species. To evaluate the protective role of Drp1 inhibition, we used three complementary approaches: gene silencing mediated by siRNA, overexpression of Drp1-dominant negative and the small molecule mitochondrial division inhibitor-1 (mdivi-1). Both morphological and functional defects induced by α-syn were attenuated by these strategies. Importantly, Drp1 inhibition reduced proteinase K-resistant α-syn aggregates. Based on that observation, we investigated the involvement of autophagy. Through a combination of stable autophagy reporter cells and immunoreactivity for LC3 and p62 in neuronal cells with either α-syn overexpression or treatment of human α-syn preformed fibrils (PFF), we observed that Drp1 inhibition abolished autophagic impairment induced by α-syn. Consistent with its role in improving autophagy function, Drp1 inhibition reduced exosome release and spread of α-syn pathology from neurons to neurons and from microglia to neurons. In summary, this study highlights new insights that Drp1 inhibition confers neuroprotection through both mitochondrial and autophagy-lysosomal pathways, further strengthening the therapeutic potential of targeting Drp1.

## Introduction

Parkinson’s disease (PD) is a complex and multifactorial disorder involving both genetic mutations and environmental factors [28]. Since the identification of the first mutation in *SNCA* [50], the gene encoding α-synuclein (α-syn), the list of additional mutations linked to PD has expanded rapidly and become rather complex [28, 29, 53]. To date, the most investigated PD-linked gene is *SNCA*. Missense mutations as well as gene duplications and triplications of *SNCA* have been identified in familial PD [3, 34, 38, 50, 61, 73]. The discovery of increasing the gene dosage of *SNCA* by two to three fold can also cause PD [61] signifies that elevated wild-type (WT) α-syn alone is sufficient to cause the disease. α-syn is prominently present in Lewy bodies, which are intra-neuronal proteins aggregates commonly observed in PD [64]. Although mutation in this gene is rare, the *SNCA* locus has been demonstrated to have genome-wide significant association with PD development [39]. Genome-wide association studies (GWAS) have identified *SNCA* as a major gene associated with sporadic PD [26, 46, 59]*.* The fact that α-syn is involved in both familial and sporadic PD makes it a significant and attractive protein to investigate pathogenic mechanisms and therapeutic target for this neurological disorder. Neurotoxic mechanisms associated with α-syn have therefore been at the forefront of the PD research and have greatly contributed to the current understanding of the disease pathology.

α-syn has been demonstrated to induce neurotoxicity through multiple but non-mutually exclusive mechanisms [7, 17, 22, 28], including impairment in mitochondrial and autophagy-lysosomal function resulting in protein aggregation, mitochondrial impairment, oxidative stress and exosome release – all of which are the topics of interest in the present study. Relevant to this study we recently published data demonstrating that by using the small molecule Mitochondrial Division Inhibitor-1 (mdivi-1), a putative inhibitor of the mitochondrial fission Dynamic-Related Protein-1 (Drp1), we were able to reduce neuropathology induced by α-syn-A53T in rats [4]. However, some critical questions remained from that study. First, mdivi-1 was used to block Drp1 function [4]. Although this inhibitor has been widely reported to produce effects consistent with blocking mitochondrial fission and GTPase function of Drp1 [42, 63], questions have been raised whether this inhibitor blocks Drp1 function [6]. Second, α-syn-A53T mutation was used to model PD. Given that this missense mutation is rare and responsible for a very small fraction of PD cases, the significance of that study in relation to sporadic PD needs to be validated in models with wild-type (WT) human α-syn. Third, to date, Drp1 is commonly referred to as a “mitochondrial fission” protein. However, most of Drp1 resides, not on mitochondria, but elsewhere in the cell. Indeed, a previous study estimated that only about 3% of Drp1 is localized to mitochondria under normal physiological condition [62]. Although under pathological condition, post-translational modifications such as phosphorylation of Drp1 at S616, would induce its translocation to mitochondria, a significant portion most likely still remains in the cytosol. It is critical to investigate additional protective mechanisms of this protein. The present study addresses these three issues and we report here that blocking Drp1 genetically improved neuropathological hallmarks associated with mitochondrial dysfunction and autophagy flux impairment induced by human α-syn-WT. Consistent with these mechanisms, we observed improved mitochondrial function as well as reduced α-syn aggregation and exosome release when Drp1 was inhibited.

## Materials and methods

### Cell cultures

#### Generation of stable and inducible human wild-type α-synuclein in dopaminergic neuronal cells

Stable cells with inducible α-syn-WT expression were generated by stably transfecting the rat dopaminergic neuronal cells N27 (1RB3A) [51, 52] (provided by Dr. Anumantha Kanthasamy, Iowa State University) with an ecdysone inducible system, the Complete Control Inducible Mammalian Expression System (Stratagene), as we previously described in details for the PINK1 models [15]. The cDNA of full length human wild-type *SNCA* was subcloned into the multiple cloning site. Stably transformed cells were selected and maintained in RPMI containing 10% FBS, G418 (500 μg/ml) and hygromycin (200 μg/ml). Of note, due to the potential concern that N27 cells in some laboratories have a mixed population because of extensive passaging over time, we performed immunocytochemistry and immunoblotting and confirmed that approximately 91% of our N27 cells have tyrosine hydroxylase (a dopaminergic marker) immunoreactivity (Additional file 1: Figure S1). Quantification was performed by counting DAPI with or without TH immunoreactivity.

Our initial attempt was to select inducible cells with high expression of GFP using flow cytometry. However, due to undetectable GFP intensity (which could occur for a protein expressed downstream of IRES) [43], we directly selected cells with high expression of α-syn using SMARTFLARE human SNCA-Cy3 mRNA probe (Millipore, SF-1254). This non-toxic probe was taken up by living cells through endocytosis. Once inside the cell, it recognizes and binds to human α-syn mRNA, resulting in fluorescent signal. For sorting, cells were treated with 20 μM PonA (or ethanol vehicle control) for 24 h and SMARTFLARE probe (200 nM) was added ~ 16 h prior to sorting using FACSAria II. Cells were maintained in RPMI 1640 containing10% FBS, G418 (500 μg/ml) and hygromycin B (200 μg/ml) (complete medium). The expression of α-syn upon PonA induction was confirmed using immunostaining and western blot.

#### Stable autophagy reporter HeLa cells

HeLa cells with stable overexpression of mRFP-GFP-LC3 were designed to monitor autophagy flux as previously described [32]. We have successfully used these cells to measure autophagy flux [9, 10]. These cells were maintained in DMEM + 10%FBS + G418 (100 μg/ml) + 1% penicillin/streptomycin. Of note, because we needed to performed immunostaining to identify cell with α-syn overexpression, we chose to use fixed cells instead of live cell imaging in this study. Due to the potential concern that fixation may bring back the quenched GFP signal, we directly compared the signal intensity of cells with or without PFA fixation. As seen below Additional file 1: Figure S2, GFP signal was not affected by fixation; otherwise, every single cell would have had a green signal – indicating autophagy flux is functional and the GFP signal is quenched when autophagosomes fuse with the acidic environment of the lysosomes.

#### Microglia

Primary cultured mouse microglia were prepared from post-natal day 0 (P0) newborn C57BL/6 pups as described [21]. Briefly, meninges-free cortices were isolated and trypsinized. Cells were cultured in complete DMEM-F12 with 10% FBS and penicillin/streptomycin. Murine monocyte colony stimulating factor (M-CSF, 10 ng/ml, cat. no.315–02, Peprotech, CA) was added to the medium 6 days after plating. After 15 days, the cultures were shaken (4 h; 260 rpm on a rotary shaker) to remove microglia.

#### SH-SY5Y cells and BV2 cells

Human neuroblastoma SH-SY5Y and immortalized mouse microglia BV-2 cells were cultured in DMEM containing 10% FBS, penicillin G and streptomycin sulfate.

### siRNA-mediated Drp1 knockdown

Pre-designed siRNA against rat *dnm1l* and human *DNM1L* (gene encoding Drp1) were purchased from Dharmacon Research, Inc. SMARTpool: siGENOME Rat *Dnm1l* siRNA was used for the N27 cells and SMARTpool: siGENOME Human *Dnm1l* siRNA was for HeLa cells. Each of this product is a mixture of four individual siRNA duplexes that target four separate sequences of the gene to maximize efficiency of gene silencing. To enhance transfection efficiency, an “in-tube” transfection procedure [15] was used with the following modifications: Cell suspension (80,000–100,000cells/ml) were mixed with jetPRIME™ DNA and siRNA Transfection Reagent (Polyplus-transfection®SA). For every 500 μL of cell suspension (RPMI + 10% FBS), 50 μL of JetPRIME buffer and 2 μL JetPRIME reagent were used. Cells were plated and left in transfection medium overnight, then media was changed the following day. Gene silencing efficiency was confirmed using western blot, with 10 nM of siRNA achieving 75–90% knockdown compared to scrambled control (siGENOME Non-Targeting siRNA Control Pools, Cat# D-001206, Dharmacon Inc) after 48 h.

### Preparation of human α-syn pre-formed fibrils (PFF)

α-syn monomers were obtained from the Michael J. Fox Foundation and the generation of PFF was performed according to the accompanying protocol (https://www.michaeljfox.org/files/accelerate/models/PFF%20Protocol%202017b.pdf). Briefly, the frozen aliquot was thawed on ice, centrifuged at 15,000×*g* for 10 min at 4 °C with a SORVALL legend micro 21R centrifuge (Thermo Scientific). Protein concentration was determined using BCA assay (Thermo Scientific Pierce) and the samples were diluted in sterile PBS to 5 mg/ml in a 1.5 ml Eppendorf protein low-bind tube. The sample was quickly centrifuged and placed in an Eppendorf ThermoMixer C orbital shaker (with thermo top on), shaken at 1000 RPM for seven consecutive days at 37 °C. The stock samples were aliquoted and stored at − 80 °C. To assess morphology of PFF, transmission electron microscopy (TEM, Phillip CM120) was used. To this end, the recombinant protein was diluted into 1 mg/ml in dPBS and sonicated using either QSonica XL-2000 at power level 2 for a total of 30 pulses (1 s each) or Fisher Scientific 120 Sonic Dismembrator equipped with CL-18 microtip (20% power) and then transferred to carbon-coated 200-mesh copper electron microscopy grids separately. Subsequently, PFF was negatively stained with 1% uranium acetate and its morphology was identified by TEM.

### PFF treatment in cell cultures

The frozen aliquot of α-syn as described above was thawed at room temperature, diluted to 0.1 mg/ml using sterile dPBS (volume 200-400 μl), and sonicated as described above. The sonicated solution was diluted to working concentrations in cell culture media prior to being used for the following experiments:

1. *SH-SY5Y* and *BV-2* cells were transfected with siRNA-Drp1(50 nM) or scramble control for 12 h, followed by PFF (2 μg/ml) treatment for 24 h, and cultured for additional 36 h after PFF withdrawal. For mdivi-1 treatment, PFF was added concurrently with mdivi-1 (20 μM). Conditional media (CM) were then collected for exosome isolation.
2. *Mouse primary microglia* were treated with PFF for 24 h in the presence or absence of mdivi-1, and further cultured for 36 h after PFF withdrawal. To activate microglia, cells were treated with LPS (1 μg/ml) for 3 h followed by 15 min of ATP (5 mM) before harvesting. Conditioned media (CM) were collected for exosome isolation.
3. *Exosomes*. Cultured cells were maintained in media containing exosome-depleted FBS (cat# EXO-FBS-50A-1, SBI System Biosciences). Mouse primary microglia were treated with PFF for 24 h in the presence or absence of mdivi-1, and further cultured for 36 h after PFF withdrawal. To activate microglia, cells were treated with LPS (1 μg/ml) for 3 h followed by 15 min of ATP (5 mM) before harvesting. Conditioned media (CM) were collected for exosome isolation. Cell culture media (20 ml) from two 100 mm plates of primary microglia was collected 24 h after PFF removal, centrifuges at 3000×*g* at room temperature for 15 min to remove cell debris. Exosomes in the resultant supernatant was precipitated using ExoQuick-TC PLUS Exosome Purification Kit (Cat# EQPL10TC-1, SBI System Biosciences) according to the manufacturer’s instructions. Exosome pellets were re-suspended in culture medium for cell treatment. To visualize exosomes, pellets of exosomes were re-suspended in sterile water and transferred to carbon-coated 200-mesh copper electron microscopy grids and incubated for 10 min at room temperature. Exosomes were then incubated with 2% phosphotungstic acid for 3 min. Micrographs were observed under a transmission electron microscope (Phillip CM120).

### Cell transfection

Cells were transfected with plasmids (0.3 μg/well for 24-well plate and 1.5 μg/well for 6-well plate) using either Lipofectamine™ 3000 (Thermo Fisher Scientific) or jetPRIME™ DNA and siRNA Transfection Reagent (Polyplus-transfection®SA) following the manufacture’s protocol.

### Immunofluorescence

Cells were grown on borosilicate cover slips pre-coated with poly-D-lysine in 24-well plates. Prior to immunostaining, cells were fixed with 4% formaldehyde (Thermo Scientific™ Pierce™, #28906), in warm cell culture media at 37 °C for 20 min. Please refer to the table in Additional file 1 for a list of primary antibodies and dilutions used. Corresponding Alexa Fluor®(350, 488, 586, and 633) conjugate secondary antibodies (Molecular Probes) were used at 1:500–1:1000 dilution. Slides were mounted using Prolong™ gold anti-fade mount with or without DAPI (Molecular Probes). Images were captured using Olympus Fluoview 1200 confocal microscope (with the exception of live cell imaging, which was Olympus Fluoview10i automated confocal laser-scanning microscope).

### Immunoblotting

For cytosolic samples, cells were lysed with RIPA buffer (150 mM NaCl,1%(v/v) IGEPAL, 0.5% Sodium Deoxycholate, 0.1% SDS, 50 mM Tris, pH 8.0) containing 1X Halt Protease and Phosphatase Inhibitor (Thermo Scientific), dounced for 20–30 times in a 1 mL glass homogenizer and centrifuged at 16,000×*g* for 15 min at 4 °C. Supernatants were collected and 30-45 μg of protein per well (depending on respective antibodies) were separated in SDS-PAGE.

For experiments related to exosome release, cells were sonicated in RIPA buffer. To measure the amount of exosomes release, exosomes were extracted from same amount of culture medium and immunoblotted for exosomal markers (Alix and Tsg101). To quantify the levels of α-syn in exosomes, the same amount of protein from exosomes (40 μg) were loaded. Proteins were separated by SDS-PAGE and then transferred onto nitrocellulose membranes. The blots were then incubated overnight at 4 °C with following primary antibodies: anti-Alix, anti-Tsg101, and anti-alpha synuclein. Secondary antibodies conjugated with horseradish peroxidase (HRP) were used, and immunoreactivity was visualized with chemiluminescence (SuperSignal Ultra, Pierce, Rockford, IL,USA). Protein bands were analyzed and quantified using Scion Image system (Scion Corporation).

### Mitochondrial morphology

N27 stable cells were grown on poly-D-lysine-coated glass coverslips. Mitochondria were visualized with transfected DsRed-Mito as we previously described [15] and images were captured using Olympus Fluoview 1200 confocal microscope. Mitochondrial morphology was quantified blinded using Image J [15]. More than 500 clearly identifiable mitochondria from randomly selected 30–50 cells per experiment were measured in four independent experiments. Roundness: 4 × ([Area])/(π × [Major axis]^2^). Aspect ratio is a measurement of major / minor axes [15], using ImageJ’, (https://imagej.nih.gov/ij/). Both of these values approach 1 as the particle becomes circular.

### Mitochondrial membrane potential (ΔΨm)

ΔΨm in N27 was quantified using 50 nM tetramethylrhodamine methyl ester (TMRM) as previously described [15]. As a positive control, cells were treated with 20 μM carbonyl cyanide 4-(trifluoromethoxy) phenylhydrazone (FCCP) to collapse ΔΨm. Fluorescent signal was analyzed by BD Accuri C6 flow cytometer using FL-2 channel.

### Reactive oxygen species (ROS) quantification

*Cellular ROS* was measured using the superoxide indicator dihydroethidium (DHE, Invitrogen) as described [55]. Briefly, N27 stable cells grown in 24-well plates were treated with 10 μM DHE in cell culture medium and incubated for 20 min at 37 °C. The dye was then washed, cells were trypsinized, and analyzed using flow cytometry (BD Accuri C6),. As a positive control, cells were treated with 30 μM H_2_O_2_.

*Mitochondrial ROS* was assessed using MitoSOX red (M36008, Molecular Probes). N27 stable cells were grown in 96-well plate and incubated with 2.5 μM of MitoSOX working solution (in HBSS) at 37 °C for 20 min. Cells were then washed three times with PBS and fluorescent intensity was measured using a plate reader (Biotek Synergy H1 Hybrid Multi-Mode Reader) at Ex/Em 510/595. After which cells were washed twice with PBS, incubated with DAPI (5 μg/ml) in the dark at room temperature for 10 min and washes three times before the DAPI signal was quantified using the Biotek plate reader (Ex/Em 358/461). Treatment of 50 nM rotenone, a mitochondrial complex I inhibitor, for 48 h was used as a positive control to generate mitochondrial ROS.

### Mitochondrial respiration

Mitochondrial function in live cells was assessed using the Seahorse XFe_96_ Extracellular Flux Analyzer (Seahorse Biosciences Inc). Cells were grown in the Seahorse 96-well plates overnight for attachment. On the following day, cell culture medium was washed and replaced with 175 μl of serum free assay medium (Dulbecco’s Modified Eagle’s Medium with - 5.5 mM Glucose, 1.0 mM Sodium Pyruvate, 4 mM Glutamine, 2 mM HEPES, pH 7.4), incubated in a 37 °C non-CO_2_ incubator for at least 30 min before being loaded into the analyzer. Mitochondrial respiration was measured using the Mito-Stress Test (Seahorse Biosciences Inc) as instructed by the manufacturer. Oligomycin (1 μg/ml), FCCP (0.5 μM), rotenone (1 μM) and antimycin A (1 μM) were sequentially added to cells to determine mitochondrial respiration. Each oxygen consumption rate data point refers to the mean rates during each measurement cycle, which consists of a mixing time of 30s followed by a data acquisition period of 3 min. Three data points were acquired after each injection, and four data points were recorded for basal respiration [69]. For normalization, cells were fixed with 4% PFA, followed by incubation of DAPI (80 μl of 5 μg/ml in PBS) for 10 min. The plate was then washed 3 time with PBS, and fluorescent signal DAPI for was quantified at Ex/Em 358/461 nm using the Biotak Synergy H1 Hybrid Reader.

To calculate total ATP production rate, which, in cell, is the sum of ATP generated by oxidative phosphorylation and glycolysis. Proton production, measured as the extracellular acidification rate in the XFe96 analyzer, also include two sources: (1) pyruvate to lactate^−^ conversion directly through glycolysis, and (2) CO_2_ to HCO_3_^−^ reaction in the TCA cycle indirectly from pyruvate feeding into the process. In addition, the amount of ATP produced per glucose through glycolysis and oxidative pathways differs significantly. Therefore, instead of directly comparing extracellular acidification rate (ECAR) with oxygen consumption rate (OCR) as cell glycolytic/metabolic index, we adapted the calculation methods by Mookerjee and colleagues [44] to compare the ATP produced by both pathways.

$$ Total\  ATP\  production={ATP}_{glyc}+{ATP}_{(ox)} $$

$$ {ATP}_{ox}=\left[ OCR\ (coupled)\ x\  2P/{O}_{ox phos}\right]+\left[{OCR}_{mito}\ x\  2P/{O}_{TCA}\right]\ \left(P/O\  defined\  as\  mol\  of\  ATP\  produced\  by\  per\  mol\  of\ oxygen\ atom\right) $$

$$ {ATP}_{glyc}=\left({PPR}_{glyc}\ x\  ATP/ lactate\right)+\left({OCR}_{mito}\ x\  2P/{O}_{glyc}\right) $$

$$ \left( Glycolytic\ rate\right)\ {PPR}_{glyc}={ECAR}_{tot}/ buffering\ power-\left[{OCR}_{mito}\ x\ \mathit{\max}\ {H}^{+}/{O}_2\ x\ \left({10}^{pH- pK1}\right)/\left( 1+\left({10}^{pH- pK1}\ \right)\right)\ \right] $$

$$ {OCR}_{mito}={OCR}_{tot}-{OCR}_{R/A} $$

### Quantification of autophagic vesicles

The analysis of autophagy flux in stable HeLa cells expressing mRFP-GFP-LC3 was performed as described [8]. Green vesicles represent autophagosomes because when the autophagosome fuses with the lysosomes, the pH sensitive GFP signal is quenched by the acidic environment in the lysosomes. Red vesicles are made up of both autophagosomes and autolysosomes. The number of autolysosomes was obtained by subtracting the number of green vesicles from that of the red vesicles. For analysis, cells were imaged with an Olympus Fluoview with 60x time objective, autophagosomes and autolysosomes from at least 50 cells per treatment group were counted using ImageJ. For N27 cells, autophagy blockage was assessed through quantification of LC3-mcherry puncta together with the immunostained p62 puncta.

### Proteinase K digestion

To determine the formation of aggregation in cells, Proteinase K digestion was performed. Freshly fixed cultured cells were washed 3X5min with PBS, then treated with Proteinase K (~ 0.34 U/ml, Sigma P4850). The plates were then incubated in the dark at room temperature for 10 min with gentle shaking, followed by 3X5min washes with PBS and subsequently immunostained for α-syn.

### Phospho4E-BP-1 immunoblotting

N27 stable cells were transfected with Drp1 siRNA (10 nM) or scramble control (10 nM) overnight and then induced with PonA for 48 h. Overnight rapamycin treatment (1 μM) was used as a control to inhibit mTOR. Cells were then harvested for western blotting. The phosphorylated form of mTOR substrate protein 4E-BP1 was probed using Phospho-4E-BP1 (Thr37/46) (236B4) Rabbit monoclonal antibody (Cell Signaling Technology, catalogue #2855), 1:500 dilution, and subsequently probed with goat anti-rabbit IgG HRP conjugate (Bio-rad) with 1:5000 dilution.

### Statistics

Data represent mean ± SEM. For normally distributed data, differences between means were analyzed using one-way ANOVA, followed by Newman-Keuls post hoc testing for pairwise comparison. The null hypothesis was rejected when the *p* value < 0.05.

## Results

### Drp1 inhibition reduces mitochondrial fragmentation induced by α-syn in rat dopaminergic neuronal cells

**Fig. 1:**
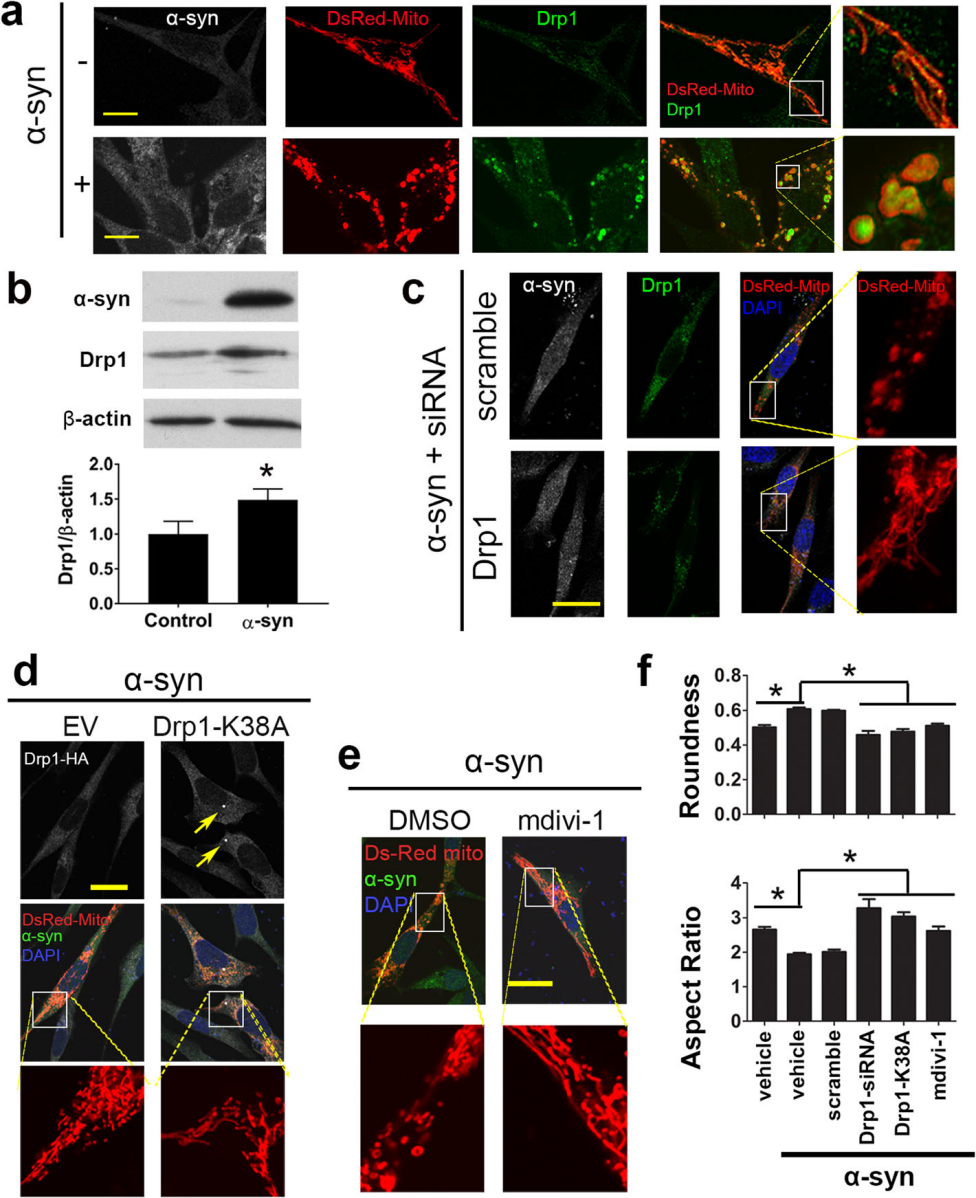
Drp1 inhibition reduces mitochondrial fragmentation induced by α-syn in rat dopaminergic neuronal cells. **a** Stable rat dopaminergic N27 cells with ecdysone-inducible system containing human wild type *SNCA* were transfected with DsRed-Mito overnight, treated with 20 μM PonA for 48 h to induce α-syn expression, followed by immunostaining for α-syn (gray) and Drp1 (green). Representative images show the effects of α-syn on mitochondrial morphology and Drp1 colocalization with mitochondria. **b** Total levels of α-syn and Drp1 in stable cells expressing empty vector control or *SNCA* after 48 h of 20 μM PonA treatment were assessed using immunoblotting. **c** N27 cells were co-transfected with DsRed-Mito and rat siRNA-Drp1 or scramble control overnight, then induced with PonA for 48 h. Representative images showing siRNA-Drp1, but not scramble control, attenuated mitochondrial fragmentation induced by α-syn. **d** As a complementary genetic approach, N27 cells were co-transfected with the dominant-negative mutation Drp1-K38A (HA- tagged) and DsRed-Mito, then induced with PonA for 48 h before immunostained for HA and α-syn. Representative images showing mitochondrial morphology in Drp1-K38A expression (arrows pointing to the characteristic Drp1 puncta formation) cells versus α-syn overexpression alone. **e** N27 cells were transfected with DsRed-Mito, and induced with PonA plus mdivi-1 (10 μM) or vehicle control (DMSO) for 48 h. Scale bars: 20 μm. Imaging data from **c**-**e** were quantified for mitochondrial morphology using Image J and summarized quantitatively in **f**. Both the values of “roundness” and “aspect ratio” approach 1 as the particle becomes circular. Data represents mean ± SEM, analyzed by one-way ANOVA (*n* = 4 or 5 independent experiments with > 500 mitochondria /group quantified for each experiment), followed by Newman-Keuls post hoc test. *p < 0.05

To have an experimental model stably overexpressing a neurotoxic protein such as α-syn, we used an ecdysone inducible system to overexpress human wild-type α-syn in the rat dopaminergic neuron cells N27. The ecdysone inducible approach provides a tight regulation of the transgene expression, a strategy that we previously used successfully to generate inducible cells overexpressing PINK1 [15]. As shown in Additional file 1: Figure S3, the cell population with inducible α-syn expression was selected by fluorescence activated cell sorting (FACS) after using a Cy3-mRNA probe specific to human α-syn mRNA. The inducible expression of α-syn was then further characterized using immunocytochemistry and western blotting (Fig. 1, Additional file 1: Figure S3). Based on our time-course and dose-response studies, a treatment of 20 μM of Ponasterone A (PonA, an ecdysone analog) for 48 h was chosen to induce α-syn expression - unless otherwise specified in some specific experiments.

After the successful generation of these stable α-syn dopaminergic neuronal cells, we first assessed the impact of α-syn on mitochondrial morphology. Using DsRed-Mito transfection to visualize mitochondria, we observed that these organelles were fragmented in cells with α-syn overexpression (Fig. 1a). However, stable cells with empty vector control displayed tubular mitochondria. This observation appeared to be mediated by Drp1 because increased levels of this fission protein were observed at the mitochondrial and total levels, as demonstrated using immunocytochemistry (Fig. 1a) and immunoblotting (Fig. 1b), respectively. To investigate the effects of Drp1 inhibition on mitochondrial fragmentation induced by α-syn, we used complementary genetic and pharmacological approaches to reduce Drp1 function as we previously described [4, 15, 56]. First, we transfected cells with siRNA-Drp1, which achieved approximately 70–80% of Drp1 knockdown efficiency (Additional file 1: Figure S4). Second, we transfected cells using the dominant negative mutant Drp1-K38A. Third, the small molecule mitochondrial division inhibitor-1 (mdivi-1). As demonstrated morphologically (Fig. 1c-e) and quantitatively (Fig. 1f), all three strategies blocked mitochondrial fragmentation induced by α-syn. Of note, siRNA-Drp1 did not appear to reduce the levels of α-syn as compared to the group that received scrambled siRNA. To quantify more objectively the levels of α-syn in these two groups of cells, we performed immunoblotting and confirmed that the levels of α-syn between cells transfected with scramble-siRNA and Drp1-siRNA were not statistically different (6.72 ± 0.31 vs 6.16 ± 1.76, data represent mean ± SEM from 3 independent experiments using actin as a loading control).

### Drp1 inhibition improves mitochondrial function and reduces oxidative stress induced by α-syn

**Fig. 2:**
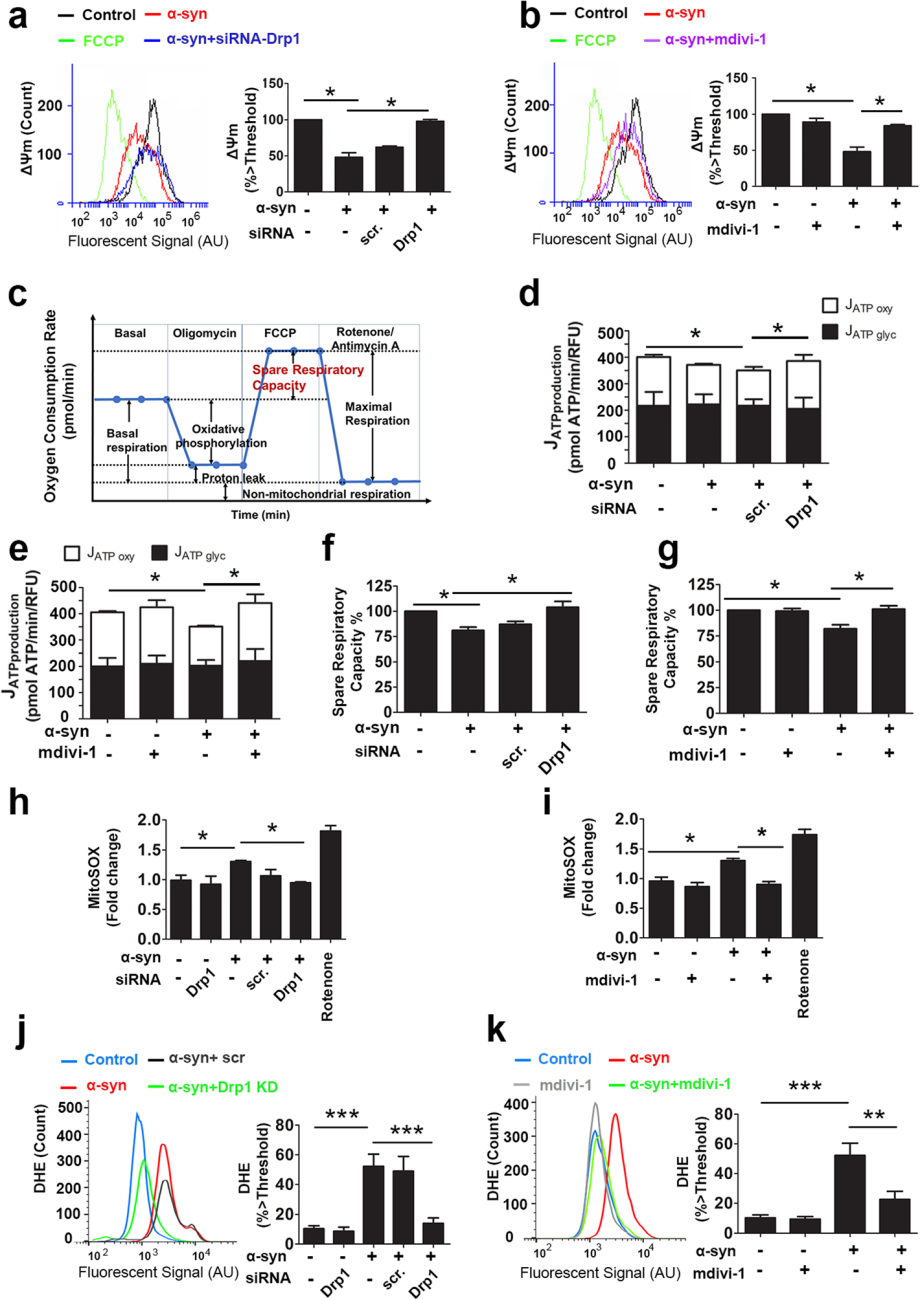
Drp1 inhibition improves mitochondrial function and reduces oxidative stress induced by α-syn. Drp1 inhibition was performed as described in Fig. 1 and the expression of α-syn was induced with 20 μM PonA for 48 h before the following experiments were performed: **a** & **b** Mitochondrial membrane potential (ΔΨm) was assessed using TMRM (50 nM), and fluorescent intensity was analyzed using flow cytometry. The uncoupler agent carbonyl cyanide 4- (trifluoromethoxy) phenylhydrazone (FCCP, 20 μM) was used as positive control to collapse ΔΨm to establish the threshold. Signal intensity (AU, arbitrary unit) was expressed as % above this threshold. **c**-**g** Mitochondrial respiration and glycolysis in live cells were assessed by measuring oxygen consumption rate and extracellular acidification rate using the XFe96 Extracellular Flux Analyzer. Sequential injections of oligomycin (to inhibit oxygen consumption mediated by ATP synthase), FCCP (an uncoupler to induce maximal OCR), Rotenone/Antimycin (to inhibit complex I and III, respectively). Spare Respiratory Capacity was calculated as % = (Maximal Respiration) / (Basal Respiration) × 100. α-syn overexpression reduced ATP production rate through oxidative phosphorylation, but not through glycolysis (**d** & **e**) as well as impaired spare respiratory capacity (**f** & **g**). Drp1 knockdown and mdivi-1 conferred protection. MitoSox red dye (**h** & **i**) and dihydroethidium (DHE, **j** & **k**) were used to measure mitochondrial and total cellular ROS, respectively, and signal intensity was quantified by plate reading and flow cytometry respectively. Data represent the mean ± SEM, one-way ANOVA (*n* = 4), followed by Newman-Keuls post hoc test. *p < 0.05 ** *p* ≤ 0.02, *** *p* < 0.001

Based on the observation that inducible α-syn-WT impaired mitochondrial morphology (Fig. 1), we asked whether mitochondrial function was also impaired in this cell model, and if so, would blocking Drp1 attenuate such dysfunction. To this end, we evaluated multiple mitochondrial function parameters. First, we measured mitochondrial membrane potential (*ΔΨ*_*m*_*)*, which is established by the electrochemical gradient from redox reactions generated by the mitochondrial electron transport chain (ETC). This gradient is responsible for driving ATP production, and therefore a decrease in *ΔΨ*_*m*_ is indicative of mitochondrial dysfunction. Using flow cytometry, we quantified fluorescent intensity of tetramethylrhodamine (TMRM) taken up by mitochondria in N27 cells. After 48 h of induction, α-syn significantly reduced *ΔΨ*_*m*_*.* Drp1 inhibition, either mediated by gene silencing (Fig. 2a) or the small inhibitor mdivi-1 (Fig. 2b), completely prevented this deficit. Second, to directly measure mitochondrial function, we quantified mitochondrial respiration using the Seahorse XFe96 Extracellular Flux Analyzer (Fig. 2c). We calculated ATP production rate by either oxidative phosphorylation or glycolysis. Figure 2d & e show that α-syn specifically reduced mitochondrial respiration but not glycolysis. siRNA-Drp1 or mdivi-1 attenuated this deficit. Next, we evaluated mitochondrial spare respiratory capacity (SRC), which represents the ability of mitochondria to provide substrate supply and electron transport in response to an increase in energy demand. α-syn suppressed SRC and siRNA-Drp1 (Fig. 2f) and mdivi-1 (Fig. 2g) preserved SRC despite the presence of α-syn.

An impairment in mitochondrial function invariably would lead to free radical production such as reactive oxygen species (ROS). To determine whether such ROS production would originate from mitochondria resulting in a higher total cellular ROS, we quantified MitoSOX Red and Dihydroethidium (DHE) signals to detect mitochondrial superoxide and cellular ROS levels, respectively (Fig. 2h-k). Rotenone, a complex I inhibitor, was used as a positive control to generate ROS from the ETC blockade. Consistent with its inhibitory effect on mitochondrial function, α-syn increased ROS levels, an adverse effect that was blunted by Drp1 inhibition (Fig. 2h-k). Taken together, these mitochondrial studies support the negative effects of α-syn on the mitochondrial ETC and that blocking Drp1 is protective.

### Drp1 inhibition attenuates autophagic blockade and protein aggregation in dopaminergic N27 neuronal cells with inducible α-syn

**Fig. 3:**
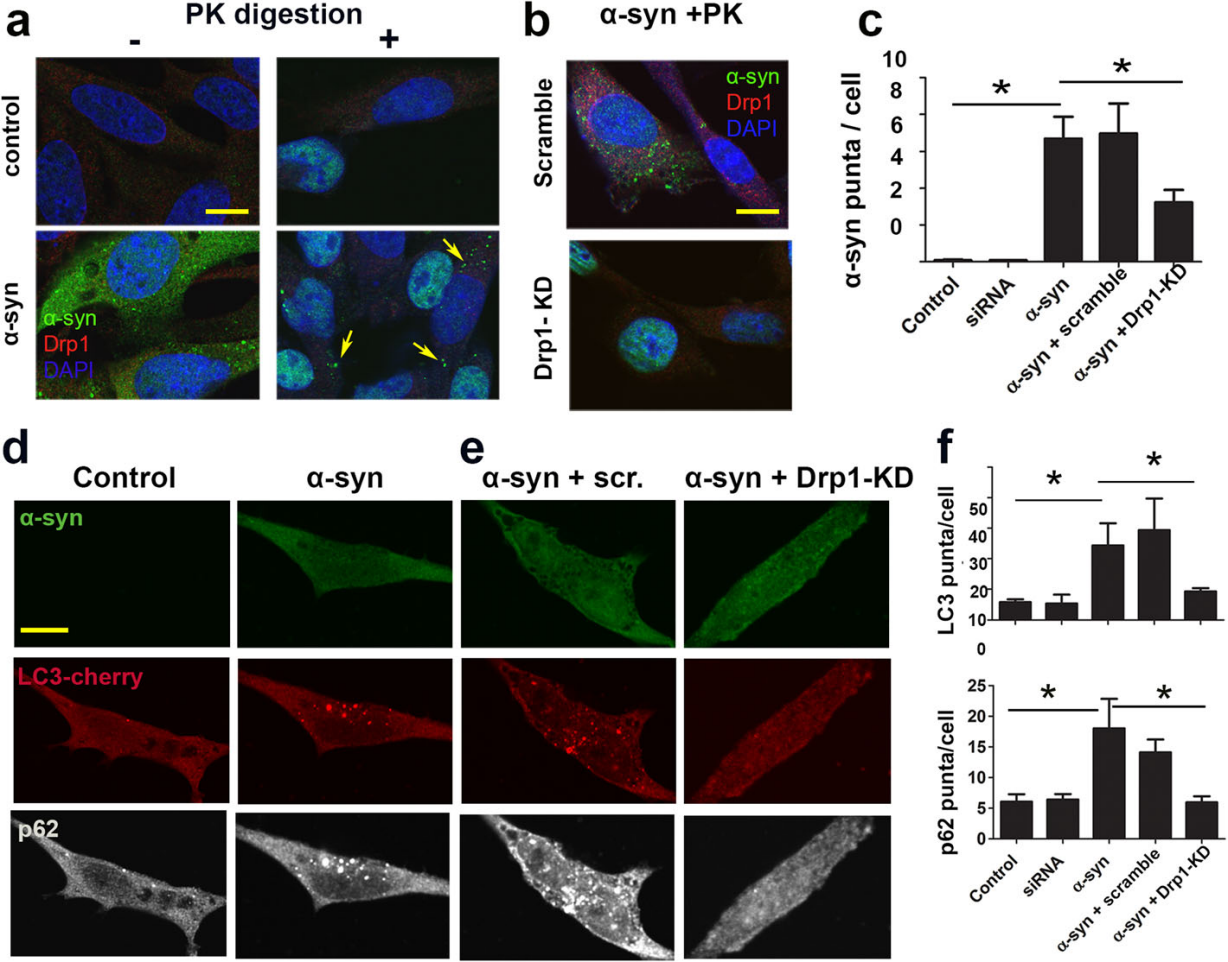
Drp1 inhibition attenuates autophagic blockade and protein aggregation in dopaminergic N27 neuronal cells with inducible α-syn. **a** & **b** Stable N27 cells were transfected with siRNA-Drp1 or scramble control for 24 h, then induced with PonA for an additional 48 h, fixed and immediately incubated with Proteinase-K (PK) for digestion. Drp1 and α-syn were then immunostained (arrows pointing to the characteristic PK resistant α-syn puncta) **c** PK resistant α-syn punta were quantified using Image J. **d** & **e** Stable N27cells were co-transfected with LC3-cherry plus either siRNA-Drp1 or scramble control for 24 h, then induced with PonA for an additional 48 h. Cells were then immunostained for α-syn and p62. **f** LC3 and p62 punta were quantified using Image J. Data represents mean ± SEM, *n* = 3–4 independent experiments with 20–30 cells per treatment group were counted in each experiment, analyzed by one-way ANOVA, followed by Newman-Keuls post hoc test. *p < 0.05. *p < 0.05 Scale bar: 10 μm

Protein aggregation is a common pathological feature of α-syn. In our cell model proteinase-K resistant α-syn aggregates were detectable 2 days after gene induction (Fig. 3a, arrows). Knocking down Drp1 with siRNA drastically reduced such protein aggregation (Fig. 3b, c). This genetic approach provided data consistent with our previous publication where α-syn-A53T accumulation in rats was significantly reduced by mdivi-1 [4]. Because autophagy is a primary pathway by which α-syn is degraded [67], one possible mechanism by which Drp1 inhibition reduced protein aggregation is by improving autophagic flux. Since LC3-II is selectively associated with autophagosomes, LC3-II or LC3-decorated vesicles has been widely used to indicate autophagosome levels or contents in cells [33]. The levels of p62 as a selective autophagy substrates inversely correlate with autophagic flux [5]. To test the hypothesis Drp1 inhibition would attenuate autophagic impairment induced by α-syn, we quantified the levels of LC3-II and p62 levels in the N27 cells with inducible α-syn expression. To achieve this objective, we transfected N27 cells with either siRNA-Drp1 or scramble control for 24 h and then induced α-syn for 48 h, followed by immunoblotting for p62and LC3I/II. The levels of p62 were significantly elevated after α-syn induction. siRNA-Drp1, but not scramble-siRNA, significantly reduced the levels of p62 in cells with α-syn overexpression but did not change the baseline levels of p62 in cells without α-syn overexpression (Additional file 1: Figure S5). Of note, endogenous levels of LC3 in N27 cells were too low to be detected reliably. Therefore, we co-transfected N27 cells with LC3-cherry plasmid to facilitate quantification of LC3 puncta in these neuronal cells. As seen in Fig. 3d-f, α-syn significantly increased the number of LC3 puncta, supporting accumulation of autophagosomes. Using immunocytochemistry to simultaneously detect p62 in these cells, we also observed an increase in p62 puncta levels, indicating a blockade of autophagy. (Fig. 3d-f). These data suggest that autophagy-lysosomal pathway is compromised in α-syn-expressing cells, consistent with previous reports [16, 23]. The accumulation of these autophagic proteins, however, was significantly attenuated by siRNA-Drp1 (Fig. 3d-f), but not scramble siRNA control, suggesting that Drp1 inhibition restores autophagy-lysosomal activity or autophagic flux.

### Drp1 inhibition prevents autophagy flux impairment induced by α-syn in autophagy reporter HeLa cells

**Fig. 4:**
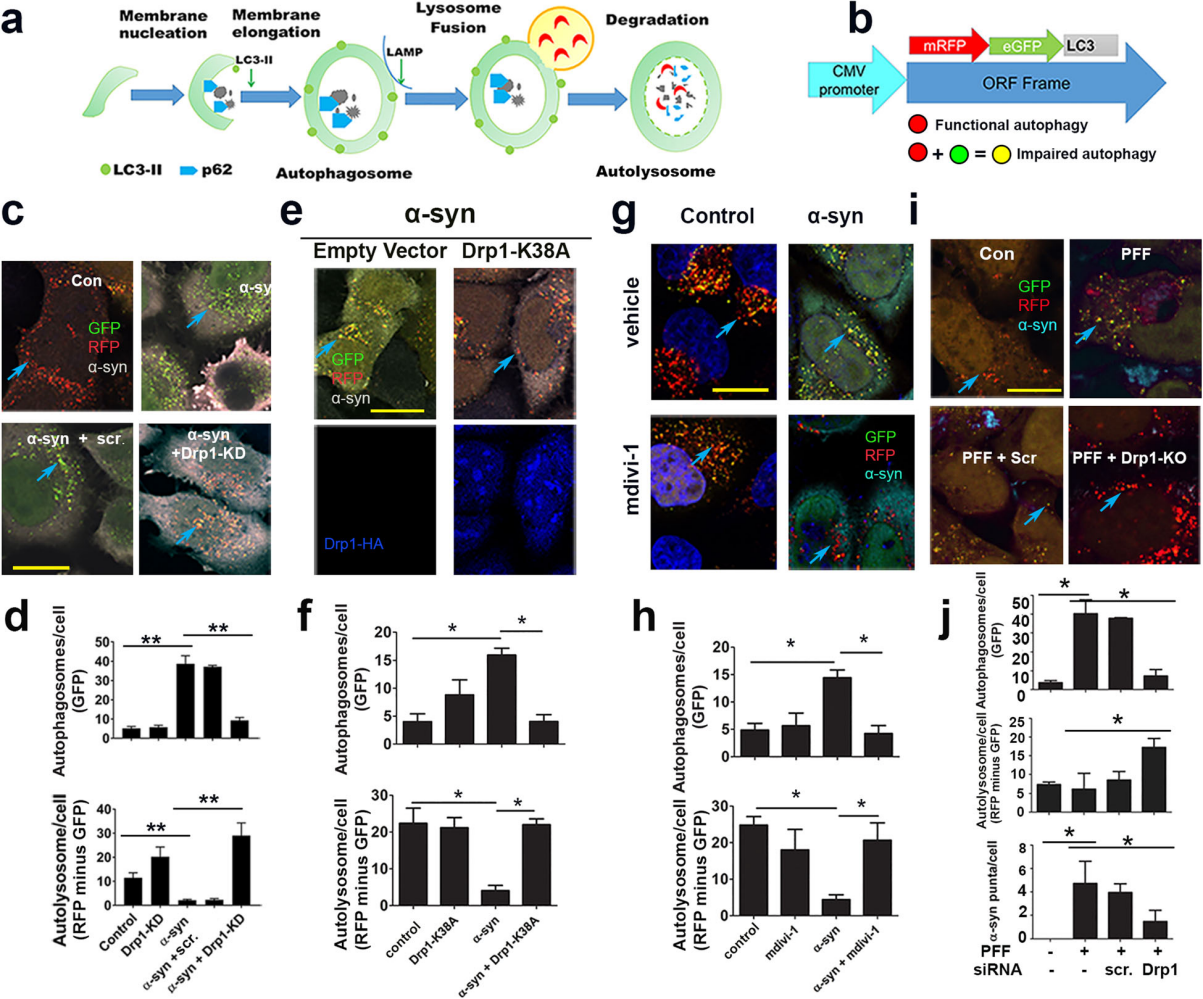
Drp1 inhibition prevents autophagy flux impairment induced by α-syn in the autophagy reporter cells. **a** Schematic diagram illustrating the autophagy flux pathway and **b** the construct used to create the mRFP-GFP-LC3 stable reporter HeLa cells. With this cell model, autophagosomes appear yellow due to the colocalization RFP and GFP signals. Red signal indicates the flux is functional because the green signal is quenched by the acidic environment of the lysosome, which fuses with autophagosome. **c** These stable Hela cells were co-transfected with human α-syn-wild type plasmid and siRNA-Drp1 or with scramble (scr) control. Representative images of cells transfected with empty vector (EV) control, α-syn, α-syn plus scramble siRNA, and α-syn plus siRNA-Drp1 were captured using confocal microscopy. **d** The numbers of autophagosomes (green vesicles) and autolysosomes (red vesicles minus green vesicles) were quantified using ImageJ. **e** & **f** As a complementary genetic approach, these reporter cells were co-transfected with plasmids expressing α-syn plus either Drp1- K38A (HA-tagged) or empty vector control. After 48 h, cells were fixed and immunostained with anti-α-syn and anti-HA antibodies and subsequently quantified for autophagosomes and autolysosomes. **g** Hela cells were transfected with α-syn as described above and treated with the putative Drp1 inhibitor mdivi-1 (10 μM) or vehicle control 24 h later. Next day, cells were fixed and immunostained for α-syn. **h**. Quantitative analysis of autophagosomes and autolysosomes using Image J. **i** Cells were transfected with either scramble or siRNA-Drp1 for 24 h before the addition of α-syn pre-formed fibrils (PFF, 8 μg/well*)* for 48 h, changed media for 24 h and then fixed and immunostained for α-syn. **j** Quantitative analysis of autophagosomes, autolysosomes and α-syn puncta was performed using image J. All data represent mean ± SEM, *n* = 3–4 independent experiment with ~ 30 cells analyzed per group, using one-way ANOVA followed by the Newman--Keuls post hoc test. *p < 0.05. Scale bar: 20 μm

As illustrated in our schematic diagram, autophagy plays a critical role in removing misfolded proteins (Fig. 4a). An impairment in autophagy has been well-established to result in accumulation of protein aggregation [57]. We recently demonstrated that pharmacological blocking of Drp1 dramatically reduced α-syn aggregates in nigral DA neurons of rats overexpressing α-syn [4]. However, it was not determined whether such protective effect was mediated through autophagy. Although data presented in Fig. 3d-f support the role of Drp1 in autophagy, to more directly monitor autophagy flux in the present study, we utilized the autophagy reporter HeLa cells with stable overexpression of mRFP-GFP-LC3 (Fig. 4b). These cells were designed to monitor autophagy flux [32]. mRFP-GFP-LC3 vesicle analysis allows us to monitor autophagosome synthesis and autophagosome-lysosome fusion by labelling autophagosomes (green and red) and autolysosomes (red), since the low lysosomal pH quenches the GFP signal. Previously, α-syn was shown to impair autophagic flux with increased autophagosome accumulation and reduced autophagosome-lysosome fusion [23]. To investigate whether blocking Drp1 would improve autophagy flux impaired by α-syn, we co-transfected these autophagy reporter cells with wild type human α-syn in the presence or absence of siRNA-Drp1 or siRNA-scramble control (Fig. 4c). After 48 h, cells were immunostained for α-syn and the number of autophagosomes and autolysosomes were quantified in these immunoreactive cells in a blinded manner. As demonstrated in Fig. 4c & d, Drp1 knockdown, but not scramble control, significantly attenuated autophagosome accumulation and enhanced autolysosomal levels in the cells with α-syn overexpression, indicating that Drp1 knockdown alleviates autophagic impairment induced by α-syn. Using another genetic approach to reduce Drp1 function, we transfected cells with the mutant Drp1-K38A dominant negative (Fig. 4e & f). In cells with Drp1-K38A expression as evidenced by immunostaining of the HA-tag, autophagy flux was significantly improved despite the co-transfection of α-syn. As a comparison to genetic approaches, we also assessed the effects of the small molecule mitochondrial division inhibitor-1 (mdivi-1) in this study. Figure 4g & h demonstrate that mdivi-1 also protected against α-syn-induced autophagy blockade.

Human α-syn preformed fibrils (PFF) has been used in recent years to induce the release of exosomes and the spread of α-syn from one cell to another in vitro [24, 40, 65] and in vivo [40]. Small seeds of PFF generated from recombinant α-syn can be endocytosed by neurons where it recruits endogenous α-syn to form phosphorylated and insoluble aggregates [65]. We obtained α-syn monomers from the Michael J. FOX Foundation and generated PFF according to the accompanying protocol. With transmission electron microscopy (TEM), we confirmed the morphology and size of PFF and its sonicated form (Additional file 1: Figure S6). Using the autophagy reporter HeLa cells, we confirmed that PFF blocked autophagy flux and siRNA-Drp1 attenuated this impairment (Fig. 4i & j). Furthermore, this treatment also reduced α-syn protein aggregation (Fig. 4i & j). Together, in a cell model designed to monitor autophagy flux, our genetic and pharmacological data provide strong evidence that blocking Drp1 alleviates the negative impact of α-syn on autophagy flux and therefore supporting a novel protective mechanism of Drp1 inhibition.

### Drp1 inhibition attenuates lysosomal impairment and inhibits mTOR activity

**Fig. 5:**
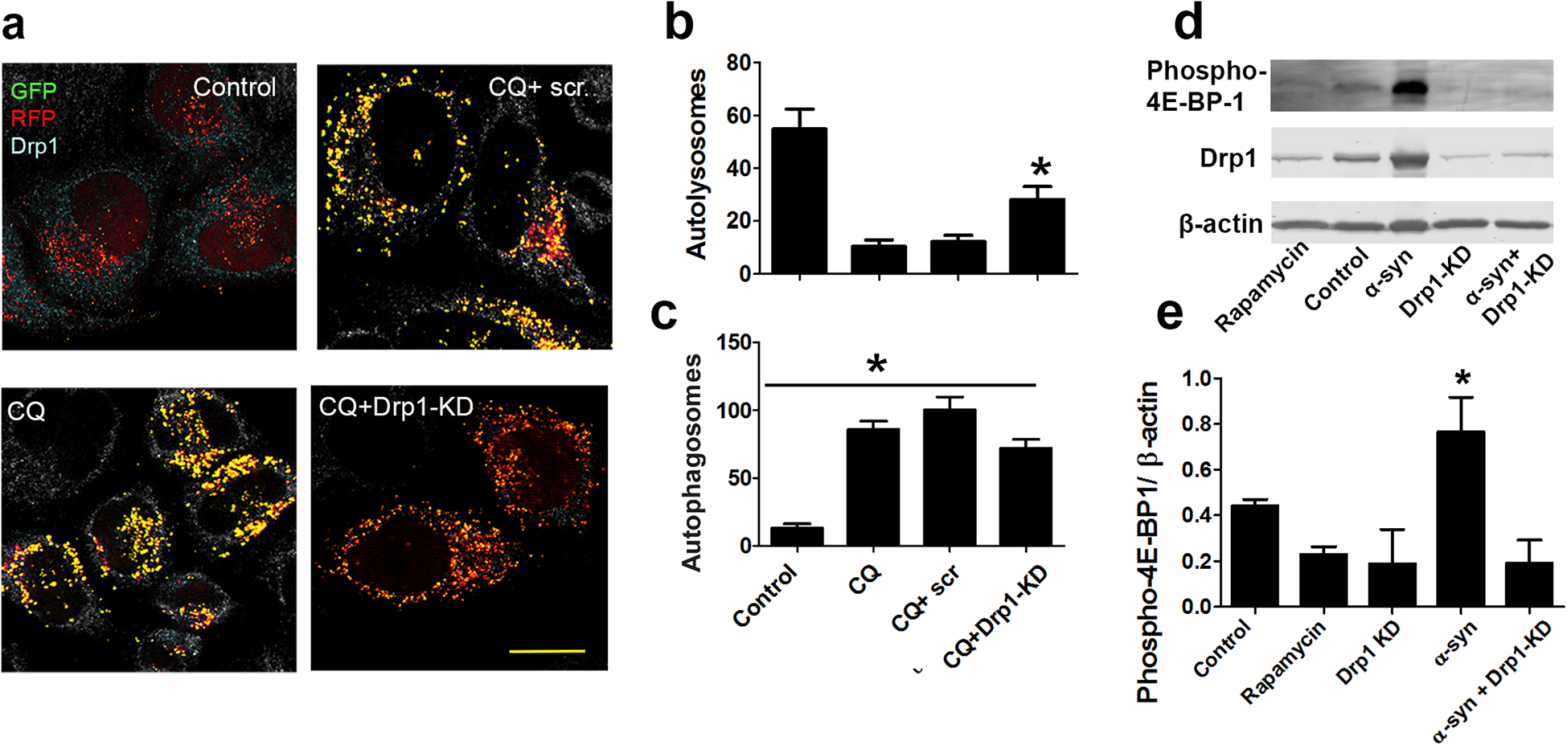
Drp1 inhibition improves lysosomal function and inhibits mTOR activity. **a - c** Hela autophagy reporter cells were transfected with siRNA-Drp1 or scramble control and then treated with 25 μM chloroquine (CQ) overnight (16 h) **a** Cells were immunostained with an Drp1 antibody. Images were captured and autophagosome/ autolysosome quantification **b** & **c** was performed using ImageJ as described above. Data represent mean ± SEM (*n* = 3 independent experiments). One-Way ANOVA with Newman–Keuls post-hoc analysis * *p* < 0.05; compared to the vehicle control group. **d** & **e** Stable N27 cells were transfected with siRNA-Drp1, and then induced with PonA (20 μM) next day to induce α-syn expression. 48 h after, cells were collected and lysed for western blot analysis **d**. Phospho-4E-BP1 was probed and normalized to β-actin **e** Data represent mean ± SEM (*n* = 4–5 independent experiments), One-way ANOVA followed by Newman–Keuls post-hoc testing * *p* < 0.05; compared to the control group

To investigate how and at what stage of autophagy flux Drp1 inhibition has an impact on, we transfected the autophagy reporter mRFP-GFP-LC3 HeLa cells (Fig. 5a) with siRNA-Drp1 or scramble control and then treated them with chloroquine to block lysosomal function, as evidenced by a reduction in autolysosomes (Fig. 5b) and an increase in autophagosomes (Fig. 5c). These alterations were attenuated by Drp1 inhibition, suggesting a partial improvement in lysosomal function, however, a direct measure of lysosome function is needed to confirm this effect. Interestingly, the number of autophagosomes remained relatively high in the cells with higher levels of autolysosomal activity under Drp1 inhibition (Fig. 5c). This result suggests that Drp1 inhibition might also promote the formation of autophagosomes. Therefore, in theory, Drp1 inhibition enhances autophagy flux by increasing both the levels of autophagosomes and the function of lysosomes. To gain additional mechanistic insights into the observed higher autophagosome levels, we transfected stable N27 cells were with siRNA-Drp1, followed by α-syn induction for 2 days and then cells were collected for immunoblotting to assess mTOR activity (which inhibits autophagy) by quantifying the levels of phosphor-4E-BP1 (Fig. 5d&e), which is a downstream substrate of mTOR. Consistent with previous study, we observed α-syn activated mTOR (Fig. 5d&e), and strikingly, knocking down Drp1 inhibited mTOR activity to an equivalent extent as rapamycin, an mTOR inhibitor. Together, our preliminary data provide a highly novel evidence that Drp1 inhibition increases autophagy flux by a combination of increasing the formation of autophagosomes and perhaps the function of lysosomes as well.

### Blocking Drp1 reduces exosome release from SH-SY5Y cells treated with PFF

**Fig. 6:**
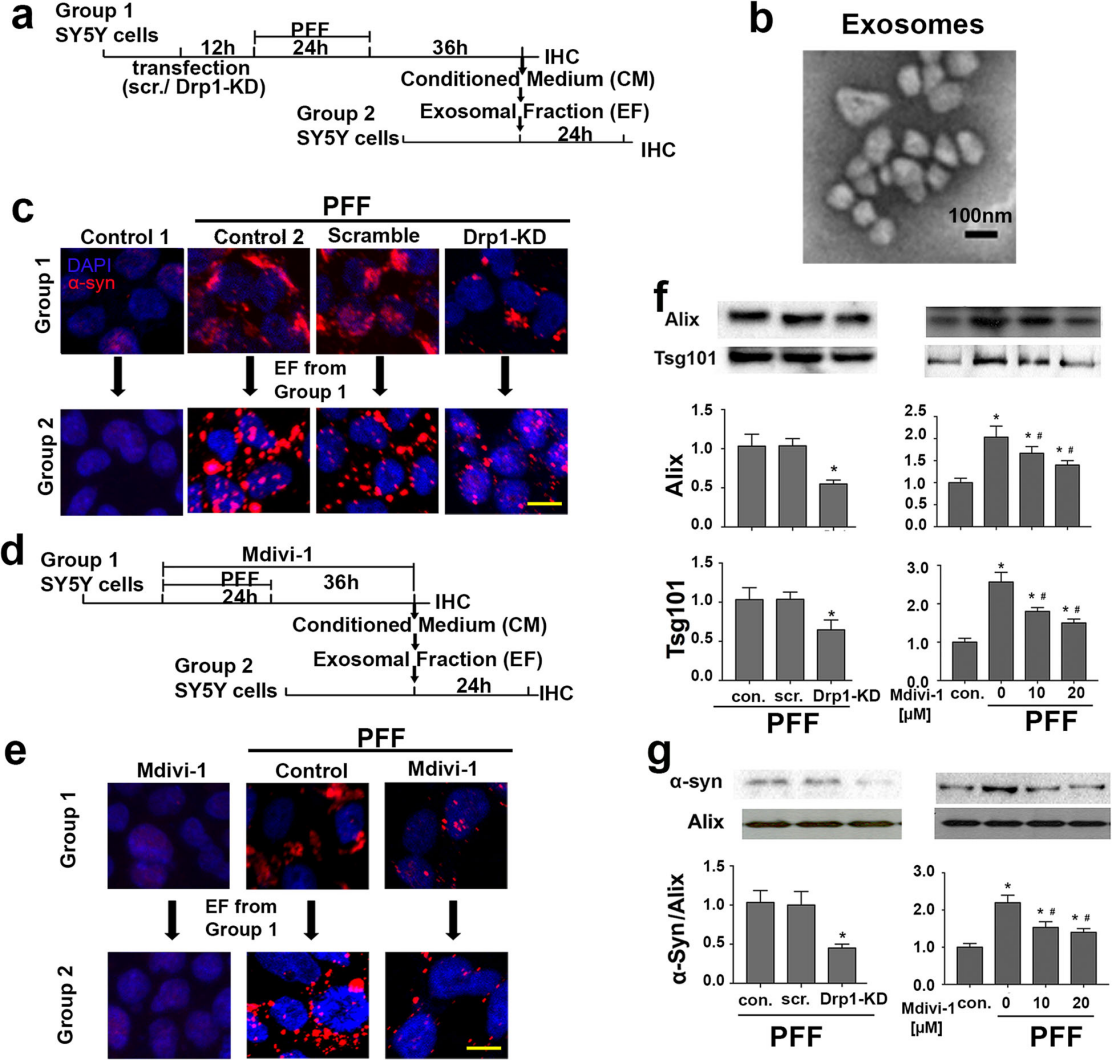
Blocking Drp1 reduces exosomal release from SH-SY5Y cells treated with PFF. **a** Experimental design: SH-SY5Y cells were transfected with siRNA-Drp1 or scramble control for 12 h, followed by PFF (2 μg/ml) treatment for 24 h, and cultured for an additional 36 h after PFF withdrawal. Conditioned media (CM) were then collected for exosomal isolation and the remaining cells were immunostained for α-syn (red). Isolated exosomes were added to a second group of SH-SY5Y cells for 24 h and then immunostained for α-syn. **b** TEM confirmed micro-vesicles in the exosomal fraction to be consistent with the size of exosomes. Scale bar: 100 nm. **c** Immunofluorescence of α-syn in two groups of SH-SY5Y cells as described in **a**: Group 1 donor cells were treated with PFF and Group 2 recipient cells were treated with the exosomal fraction extracted obtained from the Group 1 cells. Control 1 received no PFF treatment, only vehicle control. Control 2 received no siRNA. Scale bar: 10 μm. **d** Schematic diagram illustrating SH-SY5Y cells treated with mdivi-1 and **e** immunofluorescence of α-syn (red) of the Group 1 donor cells and Group 2 recipient cells as described in c. Scale bar: 10 μm. **f** Equal volume of CM was loaded into each well for immunoblotting, and the amount of exosomes were quantified using Alix and Tsg101 as markers for exosomes. Data represent the mean ± SEM, (*n* = 6). **g** To quantify the amount of α-syn release, equal amount of exosomes were used for immunoblotting, and α-syn was probed against the loading control Alix. Data represent the mean ± SEM, (*n* = 4), One-way ANOVA followed by Newman–Keuls post-hoc testing.* *p* < 0.05

Autophagosome can either fuse with lysosomes for degradation or fuse with the endosomal multivesicular bodies (MVBs) to form amphisome [60]. Upon fusion of amphisome or MVBs with the plasma membrane, exosomes are secreted as extracellular vesicles [71]. Impaired autophagy flux, therefore, would increase the release of exosomes. Based on the observations described above demonstrating that blocking Drp-1 enhanced autophagy flux and inhibited α-syn aggregation, we hypothesized that Drp1 inhibition may also reduce exosome release. Because N27 cells release undetectable levels of exosomes, we used SH-SY5Y to test this hypothesis. First, we performed Drp1 knockdown and then treated cells with PFF, followed by collecting conditioned medium and extracted exosomal fraction (EF), which was then used to treat another group of SH-SY5Y cells (see schematic diagram in Fig. 6a). Images obtained from transmission electron microscopy (TEM) revealed that EF was enriched with micro-vesicles 50–100 nm (Fig. 6b), which is consistent with the size of exosomes. Strikingly, similar to the donor cells treated with PFF (Fig. 6c), the recipient cells treated with EF for 4 days also showed strong signal for α-syn (Fig. 6c)**.** These results indicate that exosomes released from PFF treated donor neurons can be taken up by the recipient neuronal cells to serve as seeds to induce aggregation of α–syn**.** However, in cells with Drp1-knockdown, the appearance of α–syn in the donor and recipient cells was much reduced (Fig. 6c). Next, using a similar experimental paradigm (Fig. 6d), we observed that mdivi-1 also attenuated α–syn aggregation in the donor and recipient cells (Fig. 6e). To quantify the observed alterations more objectively, equal volume of conditioned medium from each group of cells was probed for the content of exosomes (Fig. 6f) and the same amount of exosomes from each group of cells was quantified for the levels of α–syn (Fig. 6g). Our data strongly indicate that not only did Drp1 inhibition reduce exosome release induced by PFF, but also less α–syn content was present in the released exosomes. These results are consistent with the mechanism of improving autophagy flux by Drp1 inhibition.

### Drp1 inhibition reduces protein aggregation induced by exosomes from PFF-treated microglia

**Fig. 7:**
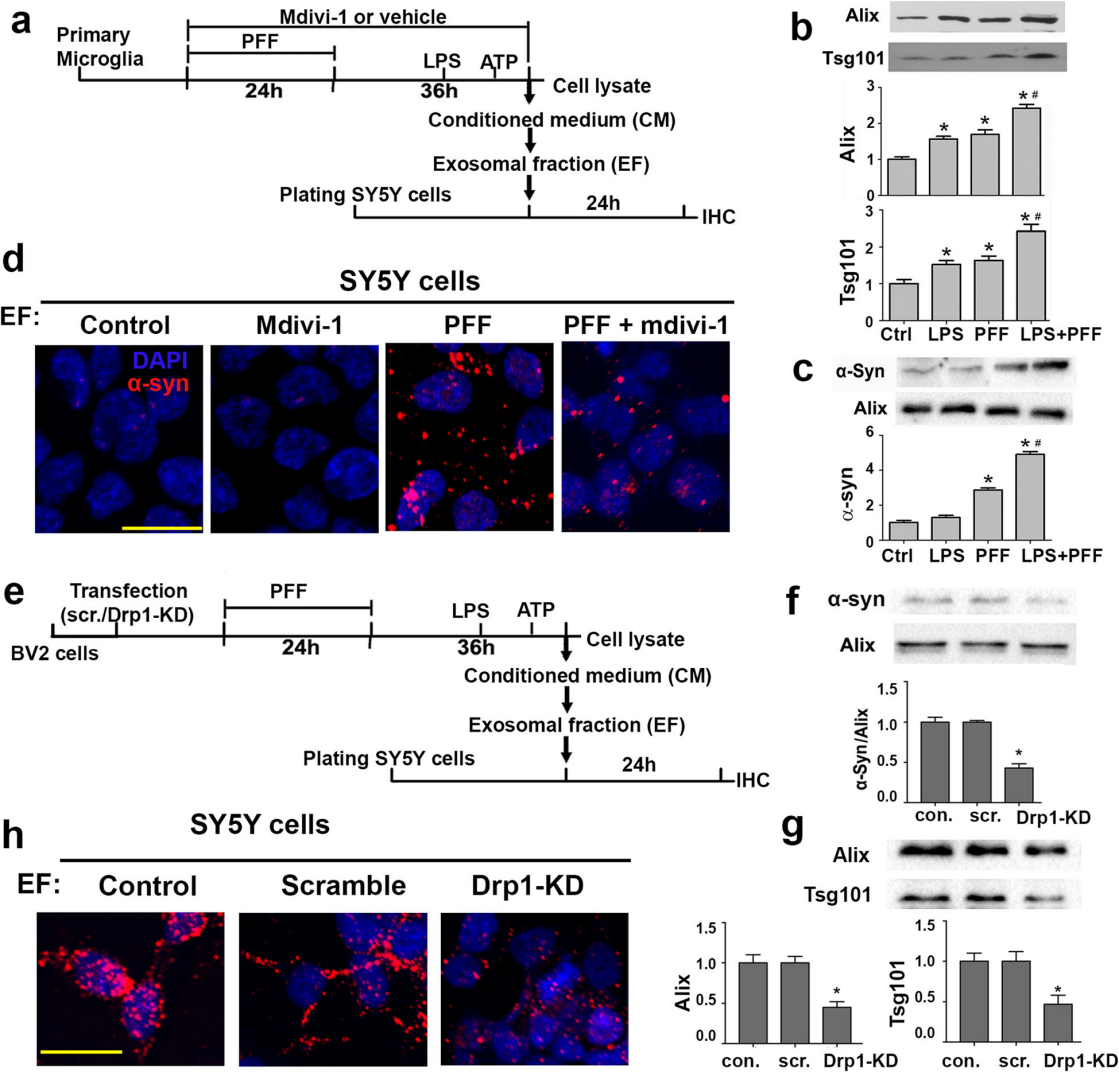
Drp1 inhibition reduces protein aggregation induced by exosomes from PFF-treated microglia. **a** Experimental scheme: In the presence or absence of mdivi-1, mouse primary microglia were treated with PFF for 24 h, and further cultured for 36 h after PFF withdrawal. To activate microglia, cells were treated with LPS (1 μg/mL) for 3 h followed by 15 min of ATP (5 mM) before harvesting. Conditioned media (CM) were collected for exosome isolation, and cells were lysed for western blot quantification: **b**. Equal volume of CM were loaded into each well and quantified for exosome levels using Alix and Tsg101 as markers. Data represent the mean ± SEM, (*n* = 3). **c** Equal amount of exosomes were loaded into each well to quantify for α-syn content. Data represent the mean ± SEM, (*n* = 4). **d** SH-SY5Y cells were incubated with extracted exosomes from PFF + LPS treated microglia for 4 days and immunostained for α-syn. Scale bar: 20 μm. **e** Experimental scheme: BV-2 microglia were transfected with siRNA-Drp1 prior to PFF treatment, and cells were treated with PFF (2 μg/ml) for 24 h and further cultured for 36 h after PFF withdraw, during which time cell were activated with LPS (1 μg/ml) and ATP (5 mM) for 3 h and 15 min, correspondingly. Then the conditional media were collected for exosome isolation, and cells were lysed for western blot quantification. **f** Same amount of EF was loaded for western blot analysis confirming the reduction of α-syn in exosomes via Drp1 silencing. Data represent the mean ± SEM, (*n* = 4). **g** Equal volume of CM were loaded into each well for western blot quantification of exosome markers Alix and Tsg101, and Drp1 silencing reduced exosome release from BV2 cells. Data represent the mean ± SEM, (*n* = 6). **h** SH-SY5Y cells treated with EF from BV2 cells were fixed after 24 h and stained for α-syn. Representative ICC images showing Drp1 knockdown in donor cells (BV2) significantly reduced α-syn aggregation formation in recipient cells (SH-SY5Y). Scale bar: 20 μm Data were analyzed using one-way ANOVA followed by Newman–Keuls post-hoc testing. * *p* < 0.05

To investigate whether microglia would release exosomes and if so, whether blocking Drp1 would attenuate such a release from microglia, we treated primary mouse microglia with LPS, PFF or both in the presence or absence of mdivi-1 (Fig. 7a). Subsequently, conditioned media were collected for exosome isolation. The amount of exosome released from primary microglia was quantified using immunoblotting (Fig. 7b). It is evident that these treatments increased the release of exosomes from microglia and when combined with LPS, PFF further enhanced the release of exosomes and their content of α-syn (Fig. 7c). To assess the spread of α-syn from microglia to neuronal cells, exosomal fraction from LPS + PFF treated microglia was incubated with SH-SY5Y cells for 4 days to allow internalization to occur. As demonstrated in Fig. 7d, confocal images revealed α-syn aggregation in these recipient neuronal cells and mdivi-1 reduced such aggregation. Due to the low level of efficiency of knocking down Drp1 in primary microglia, only the small molecule mdivi-1 was used. To further corroborate the role of Drp1 in microglial exosome release, we turned to the mouse microglial cell line BV2. These cells were transfected with siRNA-Drp1 or scramble control, followed by the PFF and LPS treatment as illustrated in Fig. 7e. Immunoblotting confirmed that Drp1 inhibition reduced the content of α-syn in microglial exosomes (Fig. 7f), reduced exosome release from microglia (Fig. 7g) and reduce the spread of α-syn to neuronal cells (Fig. 7h). In combination, results from primary microglia and BV2 cells indicate that microglia are capable of releasing α-syn-containing exosomes, thereby spreading α-syn to neurons. Blocking Drp1 significantly reduces these pathological processes.

## Discussion

Mitochondrial dysfunction and impaired autophagy flux represent two major pathogenic mechanisms in PD. After the discovery of 1-methyl-4-phenyl-1,2,3,6-tetrahydropyridine (MPTP) as the chemical causing parkinsonism [35], mitochondrial dysfunction mediated by blockade of the electron transport chain has been quite well-investigated in PD. However, a more recent approach is to understand the impact of mitochondrial fusion and fission, not only for PD but also other neurodegenerative diseases [2, 31, 66]. It is now recognized that a balance in mitochondrial fusion and fission is critical to neuronal function and viability. Mitochondrial fusion requires the coordination of both the inner (IMM) and the outer (OMM) mitochondrial membranes. The OMM proteins Mitofusin 1 & 2 (Mfn1/2) coordinate with the IMM Optic Atrophy-1 (Opa1) to join the membrane. Mitochondrial fission is governed by a separate set of proteins: Mitochondrial Fission Factor (Mff), Fission-1 (Fis1), as well as Mitochondrial Dynamics Proteins of 49 and 51 kDa (MiD49 and MiD51, respectively) are anchored to the OMM where they recruit cytosolic Dynamin-related protein-1 (Drp1), which then oligomerizes and forms a ring-like structure around the mitochondria to constrict and split them [36, 49]. Because Drp1 can bind to multiple downstream fission proteins to sever mitochondria, it has earned the reputation as a master regulator of mitochondrial fission. However, as demonstrated in the present study, in addition to this well-established function, Drp1 has a novel role in autophagy.

Autophagy plays a critical role in removing misfolded proteins, including α-syn [14, 37, 67]. Therefore, impairment in autophagy leads to accumulation of α-syn, which further exacerbating the blockade of autophagy as demonstrated cell culture and animal models [4, 11, 19, 68, 72], thus creating a bidirectional positive feedback loop of neurotoxicity [70]. Strikingly, recent data from other laboratories and ours show that blocking Drp1 is capable of reducing protein aggregation. Reddy and colleagues, for example, reported than crossing *Drp1*^*+/−*^ mice with either the AβPP (Tg2576) or the Tau P301L transgenic mouse models of (AD) reduced accumulation of toxic proteins in these animals [30, 41]. We recently demonstrated that pharmacological blocking of Drp1 dramatically reduced α-syn aggregates in nigral DA neurons of rats overexpressing α-synclein-A53T [4]. Together, these results suggest that one possible mechanism by which blocking Drp1 reduces protein aggregation is through an improvement in autophagic function.

Given the well-established function of Drp1 in mitochondrial fission and its potential role in autophagy, the present study evaluated the protective effects of Drp1 inhibition mediated through these two mechanisms. Using primarily genetic approaches to inhibit Drp1, we report here that reduced Drp1 function conferred protection against α-syn induced impairment in both mitochondria and autophagy. In stable dopaminergic neuronal cells with inducible expression of α-syn, reducing Drp1 function genetically (Drp1 knockdown and overexpression of Drp1-dominant negative) and pharmacologically (mdivi-1 treatment) attenuated the negative impacts of α-syn on mitochondrial morphology and function (membrane potential, reactive oxygen species, respiration and spare respiratory capacity (SRC). A reduction in SRC leads to energy crisis when energy demand exceeds the supply ability of mitochondria. Indeed, SRC has been considered as a major factor that defines the survival of the neuron [13]. Importantly, Drp1 inhibition drastically reduced proteinase K-resistant α-syn aggregates as demonstrated using complementary approaches of reducing Drp1 function in multiple cell models with overexpressing human wild-type α-syn or the treatment of recombinant α-syn (PFF). Consistent with these observations of protein clearance, we demonstrated that Drp1 inhibition abolished autophagic impairment induced by α-syn in multiple cell models. Furthermore, we assessed mTOR activity by quantifying the levels of its substrate, phosphor-4E-BP1, using immunoblotting of N27 cells overexpressing α-syn with siRNA-Drp1 or scramble control. These results demonstrate that reducing Drp1 function blocks mTOR activity and thereby increasing autophagy flux. In combination, our data indicate that that Drp1 inhibition confers protection against α-syn by both improving mitochondrial function and autophagy flux.

Blockade of the mitochondrial electron transport chain and autophagy flux has been shown to induce the spread of α-syn inter-cellularly. For example, exposure of enteric neurons to rotenone, a mitochondrial complex I inhibitor, promotes the release of α-syn, which is subsequently taken up by and form aggregates in the recipient neurons [47]. Emerging evidence indicates that α-syn can spread inter-cellularly through exosome release, primarily because of its ability to impair mitochondria and autophagy [1, 25]. Exosomes are small extracellular vesicles with a typical size of 40–100 nm. Because these vesicles carry cargos such as mRNA and proteins, they could play a role in the spread of misfolded proteins such as α-syn [12]. It has been demonstrated that impaired autophagy induces exosome-mediated α-syn spread to other neurons [1], forming aggregates and inducing death in the receiving cell [18, 27]. As demonstrated in this study, in addition to neurons, microglia are capable of releasing exosomes. Our data also indicate that when activated by LPS, microglia release drastically more exosomes. The role of activated microglia in causing neuroinflammation by releasing molecules such as TNF-α, IL-1β and IL-6 has been well-documented and proposed to be involved in PD pathogenesis. Relevant to this study, LPS has been reported to induce mitochondrial fission in microglia leading to neuroinflammation. Using primary microglia [45] and the BV2 murine microglial cells [48], two independent studies show that LPS induces mitochondrial fragmentation and neuroinflammation via a Drp1-dependent mechanism. Blocking Drp1 using shDrp1 and mdivi-1 reduces LPS-induced release of pro-inflammatory molecules. In addition to microglia, astrocytes are capable of causing neuroinflammation [58] - although to a lesser extent than microglia. In primary mouse astrocytes and the human U373 astrocytes, manganese (Mn) reduces oxidative phosphorylation, increases mitochondrial fragmentation and neuroinflammation - especially in the presence of supplemented aggregated α-syn [58]. The mitochondrial targeted antioxidant mito-apocynin was demonstrated to be highly protective against Mn-induced such alterations in astrocytes [58], suggesting impaired mitochondria as the cause of neuroinflammation. Together, these studies indicate that enhanced mitochondrial fission in microglia and astrocytes are linked to their production of proinflammatory molecules. It is most likely that the reduction of exosome release observed in this study was mediated through mechanisms related to improved mitochondrial function, reduced neuroinflammation and improved autophagy when Drp1 was inhibited.

## Conclusions

Mitochondrial dysfunction, impaired autophagy flux, oxidative stress and α-syn pathology (aggregation and spread) have all been proposed to play a dominant pathogenic role in PD. Blocking Drp1 function as a potential therapeutic strategy has gained interest in recent years for neurodegenerative diseases. We have reported that blocking Drp1 is neuroprotective in cell [15] and animal models of PD [4, 56]. A complementary approach using a peptide known as P110 to block the binding of Drp1 to Fis1 has also been shown to be protective in the MPTP models [20, 54]. However, all these previous studies focused on the mitochondrial fission pathway. Using models of α-syn, which is relevant to familial and sporadic PD, the present study provides the following novel observations: First, in addition to improving mitochondrial morphology and function, blocking Drp1 improved autophagy flux. Second, Drp1 inhibition reduced protein aggregation and spread from one cell to another via exosomes and non-cell autonomous mechanism. Together, these discoveries highlight new insights that Drp1 inhibition confers neuroprotection through both mitochondrial and autophagy-lysosomal pathways, further strengthening the therapeutic potential of targeting Drp1. The discovery that blocking Drp1 in microglia confers protection in neurons suggest that Drp1 should also be considered to be reduced in glial cells, not just in the affected neuronal population.

## Supplementary information

**Additional file 1.** **Figure S1.** Expression of tyrosine hydroxylase in N27 cells. **Figure S2.** Comparison of GFP signal in HeLa cells with or without PFA fixation. **Figure S3.** Generation of inducible expression of human α-synuclein in rat dopaminergic N27 neuronal cells. **Figure S4.** Efficiency of Drp1 siRNA. Figure S5 Drp1 inhibition attenuates p62 accumulation in N27 neuronal cells with inducible α-syn. Figure S6 Morphology of PFF. **Table S1.** Primary antibodies and dilutions used in western blot. **Table S2.** Primary antibodies and dilutions used in immunostaining.

## Data Availability

All data generated or analysed during this study are included in this published article and its supplementary information files.
