# Supplementary material for: Exosome release and neuropathology induced by α-synuclein: new insights into protective mechanisms of Drp1 inhibition
Source: Acta Neuropathol Commun. 2019 Nov 19;7:184. doi: 10.1186/s40478-019-0821-4 (PMC6862865; doi:10.1186/s40478-019-0821-4)
Supplement: Supplementary file 1 — Additional file 1. Figure S1. Expression of tyrosine hydroxylase in N27 cells. Figure S2. Comparison of GFP signal in HeLa cells with or without PFA fixation. Figure S3. Generation of inducible expression of human α-synuclein in rat dopaminergic N27 neuronal cells. Figure S4. Efficiency of Drp1 siRNA. Figure S5 Drp1 inhibition attenuates p62 accumulation in N27 neuronal cells with inducible α-syn. Figure S6 Morphology of PFF. Table S1. Primary antibodies and dilutions used in western blot. Table S2. Primary antibodies and dilutions used in immunostaining. [file 40478_2019_821_MOESM1_ESM.pdf]

Additional file 1

Exosome release and neuropathology induced by α-synuclein: new insights into protective mechanisms of Drp1 inhibition.

Rebecca Z. Fan[1, 2], Min Guo[1, 3], Shouqing Luo[2], Mei Cui[3,4], Kim Tieu[1,4].

[1]Department of Environmental Health Sciences, Florida International University, Miami, USA

[2]Peninsula Schools of Medicine and Dentistry, Plymouth University, Plymouth, UK

[3]Department of Neurology, Huashan hospital, Fudan University, Shanghai, China

[4]To whom correspondence may be addressed: Kim Tieu (ktieu@fiu.edu, Phone: 305 348 0371, Fax: 305-348-1996) or Mei Cui (cuimei@fudan.edu.cn, Phone: 021-52888164, Fax: 62481088)

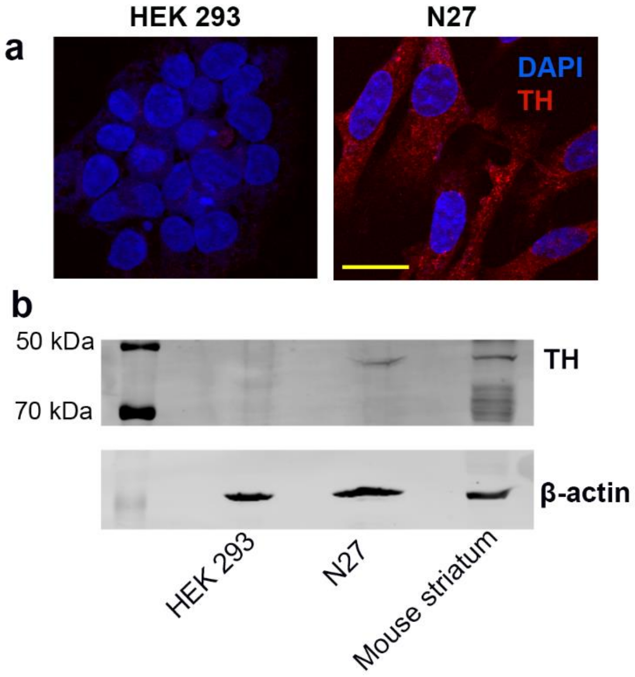

Figure S1 Expression of tyrosine hydroxylase in N27 cells. The expression of TH in N27 cells were assessed using immunocytochemistry (**a**) and immunoblotting (**b**). HEK 293 cells and mouse striatal tissues were used as a negative and positive control, respectively. Scale bar: 20 µm.

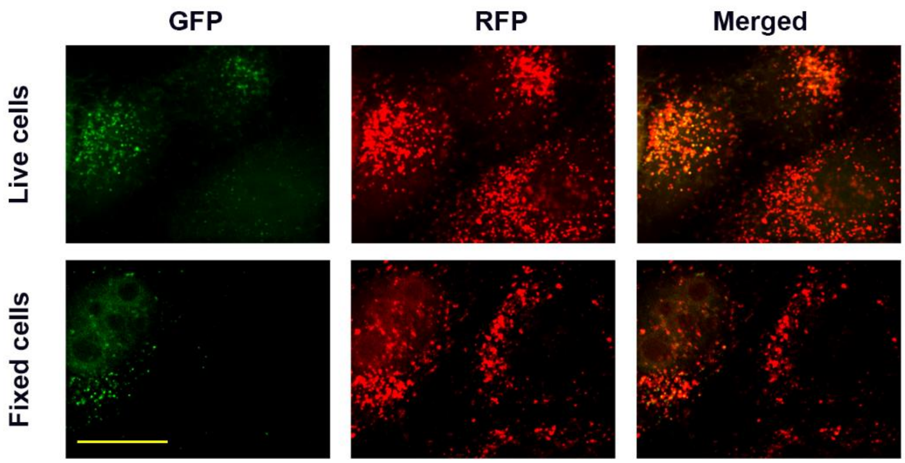

Figure S2 Comparison of GFP signal in HeLa cells with or without PFA fixation. For live cell imaging, HeLa cells were grown in Falcon® 8-well chamber slides and images were captured using Olympus FV10i Confocal Laser Scanning Microscope. For fixed cell imaging, HeLa cells were grown on borosilicate cover slips pre-coated with poly-D-lysine in 24-well plates. cells were fixed with 4% formaldehyde (Thermo Scientific™ Pierce™, #28906), in warm cell culture media at 37°C for 20 min and then images were captured using Olympus Fluoview 10i confocal microscope. Scale bar: 20 µm..

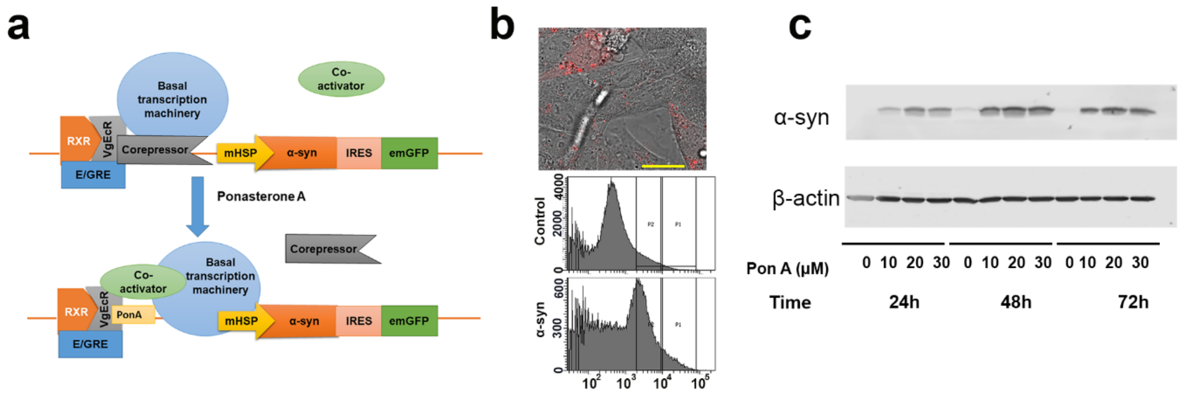

Figure S3 Generation of inducible expression of human α-synuclein in rat dopaminergic N27 neuronal cells. **a.** Based on the technology that we previously described [15], cells were first transfected with a construct constitutively expressing the ecdysone receptor (VgEcR) and the retinoid X receptor (RXR). Once these stable cells were selected, they were transfected with a second vector containing the ecdysone-responsive element (E/GRE) and a multiple cloning site with insertion of *SNCA* which encodes human wild type α-synuclein (α-syn). In the presence of ponasterone A (ponA), an ecdysone analogue inducer, the corepressors are released from VgEcR and coactivators are recruited resulting in gene activation via the minimal heat shock promoter (mHSP). **b.** Cells were induced with PonA and treated with SMARTFlare Cy3-hα-syn mRNA probe overnight before analysis. Top panel: The uptake of this probe into cells was confirmed by confocal imaging combined with phase contrast. Bottom panel: FACS was used select cells with α-syn mRNA illustrated with the shift of Cy-3 signal in the group with PonA induction. The cell population (P1) with high α-syn expression was collected and cultured for experiments. **c**. Dose-response (10, 20 and 30 µM) and time-course (1-3 days) studies of PonA were performed to determine the optimal conditions for α-syn expression. Western blot data show ponA induces α-syn overexpression in a time- and dose-dependent manner.

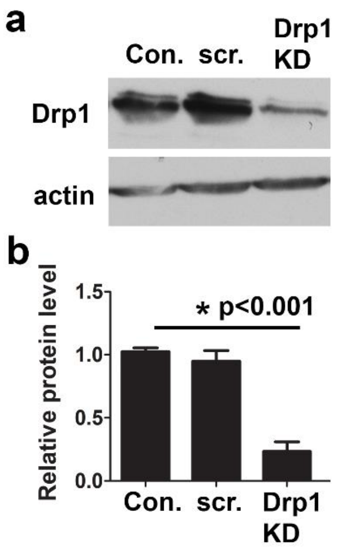

Figure S4 Efficiency of *Drp1* siRNA. **a.** N27 cells were transfected with siRNA-Drp1 or non-targeting scramble control for 48h and then collected for immunoblotting. Representative images of Drp1 levels and the loading control β-actin. **b.** Drp1 levels were normalized to β-actin and quantified using ImageJ. Data represents mean ± SEM, analyzed by one-way ANOVA, followed by Newman–Keuls post-hoc testing. n = 4 independent experiments.

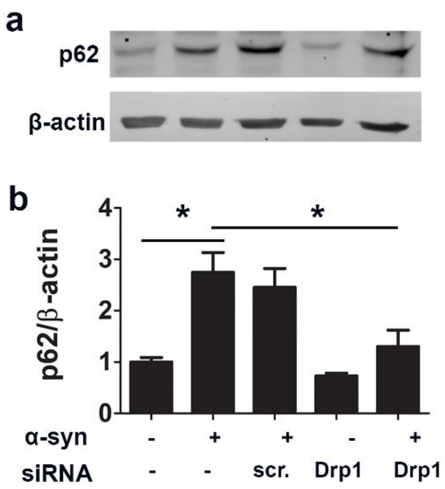

Figure S5 Drp1 inhibition attenuates p62 accumulation in N27 neuronal cells with inducible α-syn. a & b Stable N27 cells were transfected with siRNA-Drp1 or scramble control for 24h, then induced with PonA for an additional 48h, and processed for immunoblotting. p62 and β-actin (loading control) levels were quantified using Image J. Data represents mean ± SEM, n = 3-4 independent experiments, analyzed by one-way ANOVA, followed by Newman-Keuls post hoc test. *p< 0.05. *p< 0.05

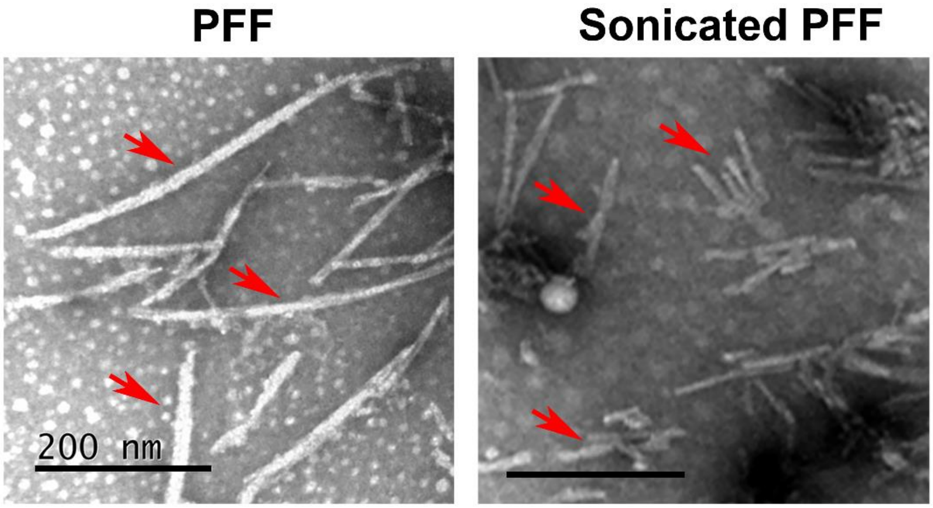

Figure S6 Morphology of PFF. Representative images of transmission electron micrographs (TEM) show morphology and size of α-syn pre-formed fibrils (PFF) before (left panel) and after sonication (right panel) as indicated by arrows.

Table 1. Primary antibodies and dilutions used in western blot

Protein target	Primary antibodies and dilutions	Secondary antibodies and dilutions
**α-synuclein**	BD Biosciences<br>#610787-Mouse monoclonal<br>1:1000	Bio-rad<br>#1706156-goat anti-mouse IgG HRP conjugate<br>1:5,000
**TH**	Novus Biologicals<br>#NB300-110, rabbit polyclonal<br>1:500	Bio-rad<br>#1706515-goat anti-rabbit IgG HRP conjugate<br>1:10,000
**Drp1**	BD Biosciences<br>#611113-Mouse monoclonal<br>1:1000	Bio-rad<br>#1706156-goat anti-mouse IgG HRP conjugate<br>1:10,000
**α-synuclein**	EMD Millipore<br>#AB5038-Rabbit polyclonal<br>1:2000	Bio-rad<br>#1706515-goat anti-rabbit IgG HRP conjugate<br>1:10,000
**ALIX**	Abcam<br>#ab117600 Mouse monoclonal [3A9]<br>1:1,000	Bio-rad<br>#1706156-goat anti-mouse IgG HRP conjugate<br>1:10,000
**TSG101**	Abcam<br>#ab125011- Rabbit monoclonal [EPR7130(B)]<br>1:1,000	Bio-rad<br>#1706515-goat anti-rabbit IgG HRP conjugate<br>1:10,000
**phospho4E-BP-1**	Cell Signaling Technology<br>#2855 Rabbit monoclonal (236B4)<br>1:500	Bio-rad<br>#1706515-goat anti-rabbit IgG HRP conjugate<br>1:5,000
**β-Actin**	Millipore Sigma<br>#A5441-Mouse monoclonal<br>1:10,000	Bio-rad<br>#1706156-goat anti-mouse IgG HRP conjugate<br>1:15,000

Table 2. Primary antibodies and dilutions used in immunostaining

Protein target	Primary antibodies and dilutions	Secondary antibodies and dilutions
**Drp1**	BD Biosciences<br>#611113-Mouse monoclonal<br>1:500	Life Technologies<br>Alexa Fluor #A11031 or #A21052-goat Goat anti-Mouse IgG (H+L) Highly Cross-Adsorbed<br>1:1,000
**HA**	Roche<br>#11 583 816 001-Mouse monoclonal (12CA5)<br>1:300	Life Technologies,<br>Alexa Fluor #A11045-Goat anti-Mouse IgG (H+L) Highly Cross-Adsorbed<br>1:500
**p62**	MBL International Corporation<br>#PM04-Rabbit polyclonal (SQSTM1)<br>1: 500	Life Technologies<br>Alexa Fluor #A21071-Goat anti-Rabbit IgG (H+L) Highly Cross-Adsorbed<br>1:1,000
**TH**	Novus Biologicals<br>#NB300-110, rabbit polyclonal<br>1:1000	Life Technologies,<br>Alexa Fluor #A11011 Goat anti-Rabbit IgG (H+L) Cross-Adsorbed Secondary Antibody,<br>1:1000
**α-synuclein**	BD Biosciences<br>#610787-Mouse monoclonal<br>1:500	Life Technologies<br>Alexa Fluor-#A11029 or #A11031 or #A11045-Goat anti-Mouse IgG (H+L) Highly Cross-Adsorbed<br>1:1,000
**α-synuclein**	EMD Millipore<br>#AB50380-Rabbit polyclonal<br>1: 2000	Life Technologies<br>Alexa Fluor #A11034-Goat anti-Rabbit IgG (H+L) Highly Cross-Adsorbed<br>1:1,000
